# Immunophenotypic skewing of B cells toward IgD⁻CD27⁻IgG⁺ subtype and metabolic attenuation in colorectal cancer

**DOI:** 10.1038/s41598-026-41446-x

**Published:** 2026-02-28

**Authors:** Eleonora Martinis, Silvia Tonon, Viviana Valeri, Alessandra Colamatteo, Carolina Ricci, Caterina Trevisan, Eleonora Capezzali, Matteo Pivetta, Serena Battista, Marta Mozzon, Alessandro Uzzau, Giuseppe Matarese, Andrea Cossarizza, Barbara Frossi, Carlo Pucillo

**Affiliations:** 1 Laboratorio di Immunologia, Dipartimento di Medicina, Università degli Studi di Udine, Udine, Italy; 2 Treg Cell Lab, Dipartimento di Medicina Molecolare e Biotecnologie Mediche, Università degli Studi di Napoli ‘Federico II’, Napoli, Italy; 3 Dipartimento di Patologia, Ospedale Universitario “Santa Maria della Misericordia” (ASUFC), Udine, Italy; 4 Present Address: Chirurgia generale, Ospedale Universitario “Santa Maria della Misericordia” (ASUFC), Udine, Italy; 5 Dipartimento di Medicina, Università degli Studi di Udine, Udine, Italy; 6 Department of Medical and Surgical Sciences for Children and Adults, University of Modena and Reggio Emilia School of Medicine, Modena, Italy

**Keywords:** Cancer, Cell biology, Immunology, Oncology

## Abstract

Colorectal cancer (CRC) is the third most prevalent cancer and understanding its tumor microenvironment (TME) is crucial for the development of innovative therapies. Despite the presence of B cells in CRC infiltrate, their clinical significance is poorly understood. In this study, we observed an enrichment of double-negative (DN) B cells, a subset lacking surface IgD and CD27, in CRC biopsies. Typically underrepresented in physiological conditions, DN B cells expand in certain chronic infections, autoimmune diseases, and cancers. Within this subpopulation, low CD21 expression—a phenotypic hallmark of exhaustion—was observed. Consistently, DN B cells displayed low metabolic activity. Accordingly, total B cells infiltrating CRC tissues showed a diminished capacity to differentiate into antibody-secreting cells (ASCs) upon stimulation. In the murine setting, CRC organoids decreased the frequency of ASCs in co-cultured B cells and induced metabolic dysfunction, marked by altered glucose and fatty acid uptake and dysregulated expression of key metabolic proteins. Moreover, B cells displayed reduced glycolysis and mitochondrial respiration, despite increased mitochondrial dependence. This study provides evidence for DN B cell accumulation within CRC infiltrate and metabolic reprogramming of B cells, suggesting that targeting B cell metabolism may represent a promising strategy to potentiate anti-tumor immune responses.

**Supplementary Information:**

The online version contains supplementary material available at 10.1038/s41598-026-41446-x.

## Introduction

Colorectal cancer (CRC) is the third most common cancer worldwide, accounting for almost 10% of all cancers. Multiple factors contribute to its onset, including high consumption of processed meats and alcohol, low intake of fruits and vegetables, smoking, and obesity. In addition to lifestyle factors, genetics can also increase risk^1^. Surgery is the standard treatment for early-stage CRC. However, in advanced stages of tumor progression, surgery is often combined with chemotherapy. Although overall survival rates for patients have improved in recent years due to modern chemotherapy regimens, these drugs are associated with a range of side effects related to their toxicity and lack of specificity, with many patients still succumbing to relapses. Additionally, drug resistance is observed in nearly all patients, limiting the effectiveness of the treatment^2^. The advent of immune checkpoint blockade therapies (ICBs) has represented a major breakthrough in the treatment of various solid tumors. However, in CRC, ICBs are beneficial for a limited subset of patients with microsatellite instability (MSI), whose tumors exhibit high mutation loads and strong immunogenicity. The vast majority of CRC cases, however, are microsatellite stable (MSS), associated with weak immunogenicity, low immune infiltration, and, consequently, poor sensitivity to ICBs^3^. The efficacy of immunotherapies is therefore influenced by the density, composition and function of immune cells within the tumor microenvironment (TME). In turn, immune cell function is continuously influenced by environmental cues, including by-products of cancer metabolism, which can modulate the metabolic profiles of immune cells, ultimately altering their functionality^4^. For example, lactate, produced in large quantities by highly glycolytic cancer cells, fuels gluconeogenesis and the tricarboxylic acid (TCA) cycle of T regulatory cells, thereby sustaining their suppressive functions^5^. Conversely, acidic environments impairs the activation and effector functions of CD8^+^ T cells, by inhibiting glycolysis^6^. The manipulation of metabolic signaling pathways in immune cells is increasingly recognized as a promising strategy to direct immune responses toward anti-tumor effects^7^. From this perspective, gaining a deeper understanding of the composition, function and metabolism of CRC tumor immune microenvironment (TIME) could support the development of more effective and comprehensive therapeutic strategies.

B cells are central components of CRC TME: they are present in tumor infiltrates, margins, and tertiary lymphoid structures in both MSI and MSS tumors^8,9^. However, their clinical significance and function remain insufficiently understood, and their metabolic behavior has yet to be explored.

This work aimed to deepen the understanding of B cell activity in CRC TME. In particular, we sought to provide an in-depth immunophenotypic and metabolic characterization of the major B cell subpopulations infiltrating this tumor, to support the identification of potential novel therapeutic targets.

## Results

### DN B cells are enriched within the B cell infiltrate of CRC

**Fig. 1:**
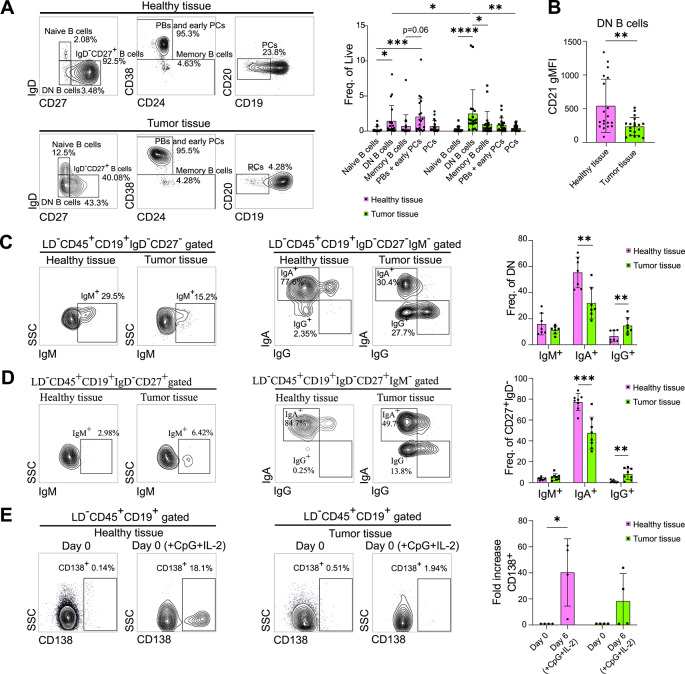
Immunophenotypic and immunoglobulin profiles of B cell subpopulations in healthy and tumor tissues.**(A)** Representative flow cytometry gates of Naïve B cells, DN B cells, Memory B cells, PBs and early PCs, and PCs infiltrating healthy (upper left panel) and tumor (lower left panel) tissues; histogram showing the percentages of each subpopulation within live cells in healthy and tumor tissues (right panel) (*n* = 20). **(B)** Histogram showing the gMFI of CD21 in DN B cells in healthy and tumor tissues (*n* = 19). **(C)** Representative flow cytometry gates of IgM^+^ (left panel), IgA^+^ and IgG^+^ (central panel) DN B cells in healthy and tumor tissues; histogram showing the percentages of IgM^+^, IgA^+^ and IgG^+^ DN B cells in healthy and tumor tissues (right panel) (*n* = 7). **(D)** Representative flow cytometry gates of IgM^+^ (left panel), IgA^+^ and IgG^+^ (central panel) IgD^−^CD27^+^ B cells in healthy and tumor tissues; histogram showing the percentages of IgM^+^, IgA^+^ and IgG^+^ IgD^−^CD27^+^ B cells in healthy and tumor tissues (right panel) (*n* = 8). **(E)** Representative flow cytometry gates showing CD138 expression on B cells isolated from healthy and tumor tissues, on day 0 and 6 days after stimulation with CpG and IL-2 (left and central panels); histogram showing the expansion of CD138^+^ B cells after 6 days of stimulation, expressed as fold induction relative to day 0 in healthy and tumor tissues (right panel) (*n* = 4). Statistical analysis was performed with Kruskal-Wallis test (panel A) and Mann-Whitney test (panels A-B-C-D-E). Data are presented as mean ± SD. *=*p* < 0.05, **=*p* < 0.01, ***=*p* < 0.001.

In order to characterize B cells infiltrating CRC, the immunophenotype of major B cell subpopulations was analyzed in tumor biopsies from stage II–III patients and compared with adjacent normal tissue (see Supplementary Table 1 for patient characteristics). Tumor biopsies were obtained from patients affected by CRC, not chemo- or radio-treated, and normal tissue was obtained by resection of a portion not less than 20 cm away from the tumor mass. Comparing the percentages of B cell subtypes within each tissue, we observed a shift in their proportions in the tumor compared to normal tissue (Fig. 1A). Specifically, while plasma blasts (PBs) and early plasma cells (PCs) represented the predominant B cell subpopulations in normal tissue, a subset of B cells lacking surface expression of both IgD and CD27 (double-negative, DN) was the most abundant within the tumor and significantly enriched compared to healthy intestine (Fig. 1A) (gating strategy in Supplementary Fig. S1).

We also revealed decreased CD21 gMFI levels in tumor-infiltrating DN B cells compared with normal tissue (Fig. 1B). IgD^-^CD27^-^CD21^low/-^ B cells represent a rare population under physiological conditions which are able to expand in chronic infections, autoimmunity and some cancers, exhibiting features of exhaustion^10–13^.

In the intestine, protection and maintenance of tissue homeostasis are primarily exerted by IgA production^14^. However, under pathological conditions, such as inflammation, the levels of antibodies can change, influencing the progression of the disease^15^. Given the imbalance of B cell immunophenotypes within the tumor, with a significant enrichment of the DN B cell subpopulation displaying exhaustion-like features (Fig. 1A and B), we sought to determine whether the immunoglobulin repertoire was also altered. We found an increased percentage of IgG^+^ cells among DN (Fig. 1C) and IgD^-^CD27^+^ B cell (Fig. 1D) subpopulations, the latter including Memory B cells, PBs and early PCs. Increased IgG levels were accompanied by decreased IgA expression on both subpopulations (Fig. 1C and D) (gating strategy in Supplementary Fig. S2). These data provide evidence for a distinct immunoglobulin repertoire of B cells infiltrating the tumor mass, characterized by an altered IgG/IgA proportion. This suggests that the TME shifts the IgA-mediated physiological response of normal intestinal B cells toward an IgG response.

Next, we investigated whether, in addition to an altered immunoglobulin signature, tumor-infiltrating B cells exhibited changes in their differentiation potential toward B cells positive for CD138, marker of antibody-secreting cells (ASCs). We sorted B cells from normal and tumor tissues, stimulated them with CpG and IL-2 and assessed ASC expansion^16,17^. The results demonstrated a significant expansion of B cells from normal tissue into CD138^+^ phenotypes following 6 days of stimulation, a response that is diminished among tumor-infiltrating B cells (Fig. 1E).

Collectively, these data suggest that tumor-infiltrating B cells are enriched in subtypes displaying exhaustion-like characteristics and show reduced responsiveness to stimuli that typically drive differentiation into ASCs.

### CRC-infiltrating DN B cells display a quiescent metabolic signature

**Fig. 2:**
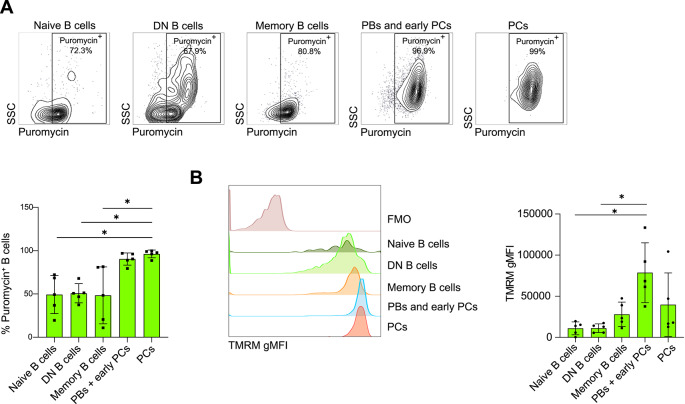
Tumor-infiltrating B cell metabolic signatures. **(A)** Representative flow cytometry gates of puromycin-positive cells within of Naïve B cells, DN B cells, Memory B cells, PBs and early PCs, and PCs infiltrating the tumor tissue (upper panel); histogram showing the percentages of puromycin-positive cells within each B cell subpopulation in the tumor tissue (lower panel) (*n* = 5). **(B)** Histograms showing the gMFI of TMRM in Naïve B cells, DN B cells, Memory B cells, PBs and early PCs, and PCs in the tumor tissue (*n* = 5). Statistical analysis was performed with Kruskal-Wallis test. Data are presented as mean ± SD. *=*p* < 0.05, **=*p* < 0.01, ***=*p* < 0.001.

Analysis of tumor tissues revealed an enrichment of DN B cells with characteristics of exhaustion and a lower responsiveness to differentiation stimuli, compared with B cells found in adjacent normal tissue. Given the close correlation between cellular function and metabolism^18^, we hypothesized that the imbalance in tumor-infiltrating B cell subpopulations -and associated functions- might be related to a dysfunctional metabolic profile. Therefore, we measured protein synthesis levels, as an indicator of metabolic activity by treating cells with the translation inhibitor, puromycin^19^. The incorporation of puromycin was detected with a specific fluorescence-conjugated antibody (Anti-Puromycin antibody) by flow cytometry, along with markers of the major subpopulations of B cells^19^. Comparable percentages of puromycin-positive Naïve, DN, and Memory B cells were observed within the tumor tissue (Fig. 2A). Consistently, the gMFI of the mitochondrial membrane potential indicator, TMRM, was similar between the three subpopulations (Fig. 2B). Overall, we showed that most tumor-infiltrating B cells, namely DN B cells, exhibit a metabolic profile similar to that of Naïve and Memory B cells, which are generally resting and metabolically quiescent, although still responsive to antigens^18^. By contrast, the low metabolic activity of DN B cells could plausibly explain the observed defects in the differentiation potential of B cells toward mature and functional phenotypes, the ASCs (Fig. 1E).

### Tumor organoids impair glycolysis in B cells

**Fig. 3:**
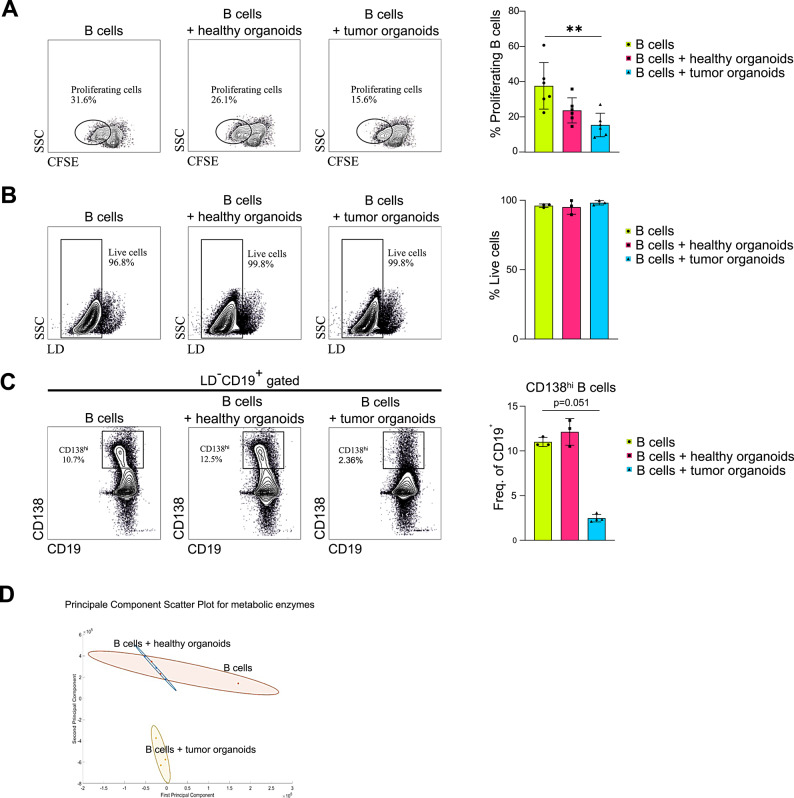
**B cell proliferation**,** viability and ASC frequencies following co-culture with organoids.** **(A)** Representative flow cytometry gates of proliferating B cells alone and after co-culture with healthy and tumor organoids (left panel); histogram showing the percentages of proliferating B cells (right panel) (*n* = 6). **(B)** Representative flow cytometry gates of live B cells alone and after co-culture with healthy and tumor organoids (left panel); histogram showing the percentages of live cells (right panel) (*n* = 3). **(C)** Representative flow cytometry gates of CD138^hi^ B cells alone and after co-culture with healthy and tumor organoids (left panel); histogram showing the percentage of CD138^hi^ B cells in each experimental group (right panel) (*n* ≥ 3). **(D)** QSM Principal Component Analysis for metabolic proteins of B cells alone and after co-culture with healthy and tumor organoids (*n* = 3). Statistical analysis was performed with Kruskal-Wallis test. Data are presented as mean ± SD. *=*p* < 0.05, **=*p* < 0.01, ***=*p* < 0.001.

Since most CRC-infiltrating B cells exhibited low metabolic activity, we investigated in vitro the molecular mechanisms underlying the observed metabolic impairment. Murine organoid lines were established from both normal intestine and Azoxymethane/Dextran Sodium Sulfate (AOM/DSS)-induced colon adenomas respectively, to recapitulate the healthy and tumor gut microenvironment^20^. Co-cultures of organoids and splenic B cells were generated by embedding the organoids in Matrigel drops, while B cells were maintained in suspension within the surrounding culture medium (Supplementary Fig. S3). The use of splenic B cells is supported by their high degree of phenotypic and functional heterogeneity, which closely mirrors that of B cells residing in secondary lymphoid structures of the healthy intestine, such as Peyer’s patches, as well as in tertiary lymphoid structures (TLS) that form in a variety of diseases, including cancer^21^. Lipopolysaccharide (LPS) was added to the medium to mimic its physiological abundance in the intestinal tract^22^. After 48 h of co-culture with tumor organoids, B cells exhibited a significant reduction in proliferation compared to B cells alone, and a slight decrease relative to those cultured with healthy organoids (Fig. 3A), while cell viability remained unaffected (Fig. 3B). Moreover, consistent with findings in human samples (Fig. 1E), B cells cultured with tumor organoids showed a reduced frequency of CD138^hi^ cells (Fig. 3C), suggesting alteration of differentiation potential.

We next investigated the metabolic profile of B cells by evaluating the expression levels of key metabolic proteins. Mass spectrometry analyses were performed using the functional proteomics technology, QSM. Principal Component Analysis (PCA) of metabolic proteins revealed that B cells alone cluster closely with B cells co-cultured with healthy organoids, indicating similar metabolic profiles. In contrast, B cells co-cultured with tumor organoids formed a distinct cluster, indicating a divergent metabolic state that may contribute to altered proliferation and differentiation (Fig. 3D).

**Fig. 4:**
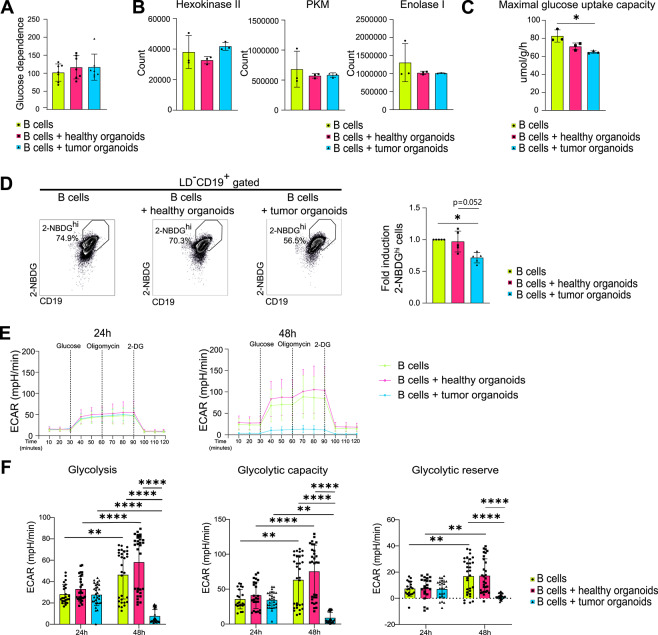
Tumor organoids impair B cell glucose metabolism.**(A)** Glucose dependence of B cells alone and after co-culture with healthy and tumor organoids (*n* = 7). **(B)** QSM protein quantification of glycolytic enzymes in B cells alone and after co-culture with healthy and tumor organoids (*n* = 3). **(C)** Maximal glucose uptake capacity of B cells alone and after co-culture with healthy and tumor organoids (*n* = 3). **(D)** Representative flow cytometry gates of 2-NBDG^hi^ B cells alone and after co-culture with healthy and tumor organoids (left panel); histogram showing the fold increase in 2-NBDG^hi^ B cells following co-culture with healthy and tumor organoids, relative to B cells alone (right panel) (*n* = 5). **(E)** Kinetic profile of ECAR in B cells alone and after co-culture with healthy and tumor organoids, after 24 (left panel) and 48 (right panel) hours (*n* ≥ 8). **(F)** Glycolysis, glycolytic capacity and glycolytic reserve of B cells alone and after co-culture with healthy and tumor organoids at 24 and 48 h (*n* ≥ 24). Statistical analysis was performed with Kruskal-Wallis test (panels A-B-C-D) or two-way ANOVA (panel F). Data are presented as mean ± SD. *=*p* < 0.05, **=*p* < 0.01, ***=*p* < 0.001.

Glucose metabolism was then assessed. First, glucose dependence was evaluated using SCENITH (single-cell energetic metabolism by profiling translation inhibition) method^19^. No differences in this parameter were observed upon 48-hour co-culture with both healthy and tumor organoids, indicating that B cells relied on glucose oxidation for ATP production at similar levels across all experimental conditions (Fig. 4A, Supplementary Fig. S4). Protein expression levels of key glycolytic enzymes Hexokinase II, PKM and Enolase I (glycolytic pathway in Supplementary Fig. S5) were measured by QSM. No differences in the expression of these proteins were observed between B cells co-cultured with healthy versus tumor organoids (Fig. 4B), and Western blot analyses confirmed similar trends (Supplementary Fig. S6-S10). QSM analysis revealed instead a reduction in glucose uptake capacity in B cells after co-culture with tumor organoids, statistically significant when compared to the condition of B cells alone (Fig. 4C). Consistent with this finding, a decreased percentage of B cells showing high levels of the fluorescent glucose analogue 2-NBDG (2-NBDG^hi^ cells) was detected after 48 h of interaction with tumor organoids (Fig. 4D), suggesting lower glucose absorption. Seahorse analyses were conducted to examine potential changes in the glycolytic function of B cells, as a possible consequence of altered glucose uptake capacity. No changes were detected in glycolysis, glycolytic capacity, or glycolytic reserve of B cells after 24 h of interaction with either healthy or tumor organoids (Fig. 4E and F). After 48 h, B cells cultured alone or co-cultured with healthy organoids exhibited an increase in glycolysis compared with 24-hour levels, likely due to the presence of LPS in culture medium. In contrast, B cells maintained with tumor organoids displayed a decrease in glycolysis over time, with similar trends observed in glycolytic capacity and glycolytic reserve (Fig. 4E and F). Overall, these data demonstrate that glycolysis is impaired in B cells co-cultured with tumor organoids, likely due to reduced glucose uptake capacity, rather than defects in the expression of glycolytic enzymes.

### Tumor organoids influence B cell mitochondrial metabolism, driving metabolic reprogramming towards greater mitochondrial dependence

**Fig. 5:**
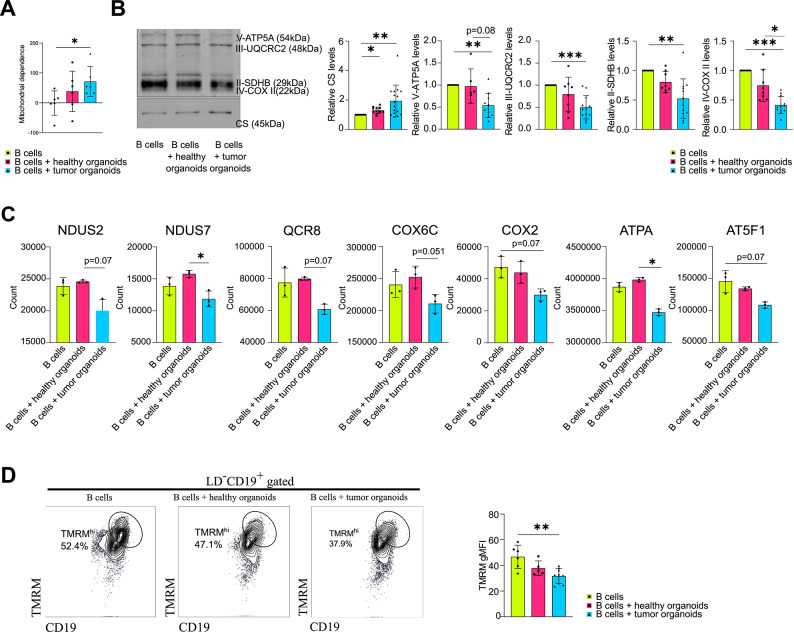
Tumor organoids alter B cell mitochondrial dependence and expression of mitochondrial proteins. (**A**) Mitochondrial dependence of B cells alone and after co-culture with healthy and tumor organoids (n=6). (**B**) Western blot analysis of CS (lower blot), ATP5A, UQCRC2, SDHB and COX II (upper blot) in B cells alone and after co-culture with healthy and tumor organoids (left panel) (cropped blots are shown; original blots are presented in the Supplementary Fig. S12-S14); histograms showing the densitometry analysis of CS calculated over Revert700 Total protein stain signal (Supplementary Fig. S11) and of ATP5A, UQCRC2, SDHB and COX II calculated over CS signal, expressed in fold induction relative to B cells alone (right panel) (n≥8). (**C**) QSM protein quantification of TCA and ETC proteins in B cells alone and after co-culture with healthy and tumor organoids (n=3). (**D**) Representative flow cytometry gates showing the percentages of TMRMhi population in B cells alone and after co-culture with healthy and tumor organoids (left panel); histogram showing the percentages of TMRMhi B cells in each experimental group (right panel) (n≥5). Statistical analysis was performed with Kruskal-Wallis test. Data are presented as mean ± SD. *=p<0.05, **=p<0.01, ***=p<0.001.

**Fig. 6:**
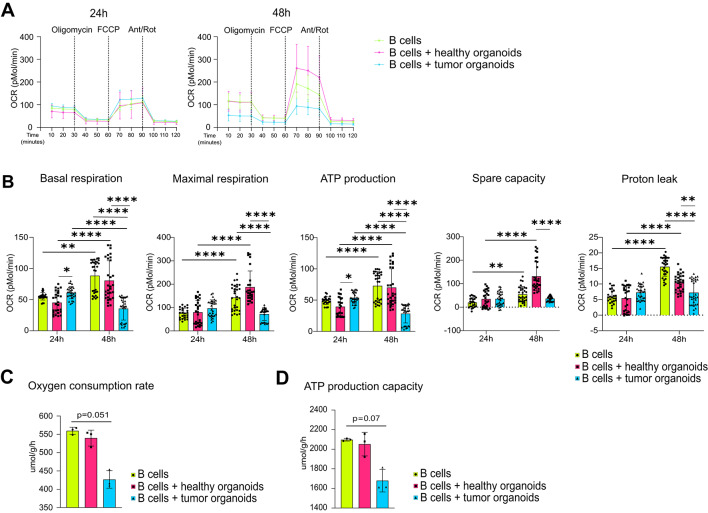
**Tumor organoids impair B cell mitochondrial function. (A)** Kinetic profile of OCR in B cells alone and in co-culture with healthy and tumor organoids after 24 (left panel) and 48 (right panel) hours (*n* ≥ 8). **(B)** Basal respiration, maximal respiration, ATP production, spare capacity and proton leak of B cells alone and after co-culture with healthy and tumor organoids, at 24 and 48 h (*n* ≥ 24). **(C)** Maximal oxygen consumption rate of B cells alone and after co-culture with healthy and tumor organoids (*n* = 3). **(D)** Maximal ATP production capacity of B cells alone and after co-culture with healthy and tumor organoids (*n* = 3). Statistical analysis was performed with two-way ANOVA (panel B) or Kruskal-Wallis test (panels C-D). Data are presented as mean ± SD. *=*p* < 0.05, **=*p* < 0.01, ***=*p* < 0.001.

Next, we assessed whether the interaction with the organoids induced an alteration in mitochondrial metabolism. By SCENITH method, we observed that B cells co-cultured with tumor organoids exhibited a significant increase in mitochondrial dependence compared with B cells alone, and a slight increase compared with those cultured with healthy organoids (Fig. 5A, Supplementary Fig. S4). We assessed whether the observed increase in mitochondrial dependence was reflected by changes in mitochondrial mass by measuring the protein expression levels of citrate synthase (CS), used as a marker of mitochondrial content^23^. We detected an augmented expression of CS in B cells co-cultured with organoids, particularly tumor organoids, for 48 h, suggesting that B cells experienced an increase in mitochondrial size or number in this culture condition (Fig. 5B, Supplementary Fig. S11). In parallel, however, we observed a reduced expression of multiple subunits of the electron transport chain (ETC) complexes, exclusively in the presence of tumor organoids. Specifically, ATP5A of complex V, UQCRC2 of complex III, SDHB of complex II (which is also a TCA enzyme) and COX II of complex IV were downregulated in this condition (Fig. 5B). Consistently, QSM analysis indicated slight or, in some cases, significant trends toward downregulation of additional ETC subunits, including NDUS2 (complex I), NDUS7 (complex I), QCR8 (complex III), COX6C (complex IV), COX2 (complex IV), ATPA (complex V) and AT5F1 (complex V) (Fig. 5C). In line with these findings, the percentage of B cells showing high levels of TMRM was significantly reduced upon interaction with tumor organoids, indicating a lower mitochondrial membrane potential (Fig. 5D). Mitochondrial respiration was assessed by measuring the oxygen consumption rate (OCR) of B cells after 24 and 48 h of co-culture. After 24 h, basal respiration and ATP production of B cells co-cultured with tumor organoids were higher than B cells with healthy organoids. After 48 h, these indices increased in B cells alone and with healthy organoids, while decreasing in the presence of tumor organoids (Fig. 6A and B). A drastic reduction in maximal oxygen consumption and ATP production capacities, at the same time-point, was also inferred from the quantitative data on mitochondrial enzymes obtained through QSM analysis (Fig. 6C and D).

**Fig. 7:**
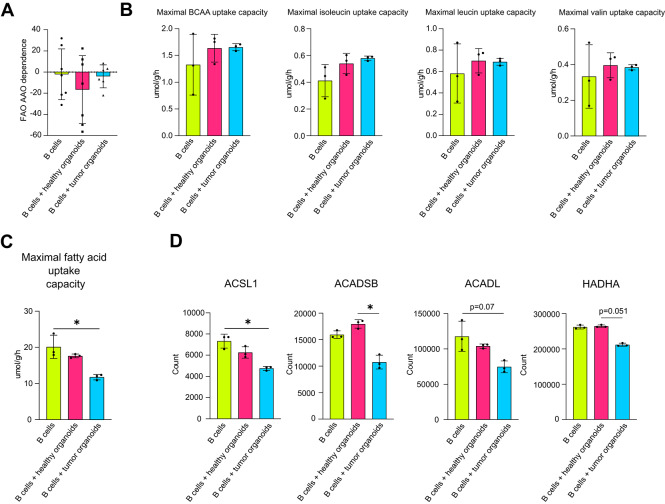
B cells undergo an impairment in lipid metabolism following interaction with tumor organoids.**(A)** AAO and FAO dependence of B cells alone and after co-culture with healthy and tumor organoids (*n* = 7). **(B)** Maximal BCAA uptake capacity of B cells alone and after co-culture with healthy and tumor organoids (*n* = 3). **(C)** Maximal FA uptake capacity of B cells alone and after co-culture with healthy and tumor organoids (*n* = 3). **(D)** QSM protein quantification of enzymes involved in lipid synthesis and degradation in B cells alone and after co-culture with healthy and tumor organoids (*n* = 3). Statistical analysis was performed with Kruskal-Wallis test. Data are presented as mean ± SD. *=*p* < 0.05, **=*p* < 0.01, ***=*p* < 0.001.

To gain deeper insights into mitochondrial metabolism, we aimed to investigate whether, in addition to the decreased production of TCA and ETC enzymes, there were alterations in the metabolic pathways that provide intermediates to replenish the TCA cycle, thereby supporting downstream oxidative phosphorylation (OXPHOS): the amino acid oxidation (AAO) and fatty acid oxidation (FAO) pathways. No differences in AAO and FAO dependence were observed by SCENITH analysis between B cells alone and those co-cultured with either healthy or tumor organoids (Fig. 7A, Supplementary Fig. S4). Similarly, no changes in branched-chain amino acid (BCAA) uptake capacity were detected by QSM between B cells co-cultured with healthy or tumor organoids (Fig. 7B). In contrast, fatty acid (FA) uptake capacity was reduced in B cells after co-culture with tumor organoids (Fig. 7C), as well as the protein levels of enzymes involved in both lipid synthesis and degradation, including ACSL1, ACADSB, ACADL and HADHA (Fig. 7D, Supplementary Fig. S15).

Overall, these results suggest that the initial interaction with tumor organoids enhances mitochondrial activity in B cells. However, B cells seem unable to sustain this increased mitochondrial metabolism over an extended period, possibly due to changes in substrate uptake and the expression of enzymes involved in the TCA cycle, FAO, and ETC. As a result, mitochondrial respiration significantly decreases at the 48-hour time point, despite the higher mitochondrial dependence.

### Discussion

B cells are widely recognized for their role in anti-tumor immune responses in various cancer types. However, in CRC, the composition and functional implications of infiltrating B cell subtypes remain largely uncharacterized and warrant further investigation. In this study, we show the enrichment within the CRC infiltrate of a subpopulation of B cells, the DN B cells (IgD^-^CD27^-^)(Fig. 1A), known to be underrepresented in physiological conditions and expanded in the blood of patients with chronic infections, autoimmunity and certain tumors^24^. DN B cells differentiate from Naïve B cells in inflammatory environments that promote exposure to disease-specific antigens, cytokines, and interleukins^25^. Consequently, their function is complex and context dependent. In Systemic Lupus Erythematosus (SLE), DN B cells have been identified as precursors to Memory B cells and ASCs, thereby contributing to disease progression^26,27^. Conversely, in HIV patients, DN B cells expressing the inhibitory receptor FcRL4—which dampens BCR signaling—exhibit dysfunction and exhaustion^12,28^. Similarly, R.A. Belderbos et al. described a subpopulation of circulating DN B cells in patients with non-small cell lung cancer (NSCLC), phenotypically identical to exhausted B cells found in chronic infections, including HIV, malaria and tuberculosis^29^, which correlate with a reduced response to checkpoint inhibitor therapy^24^.

Our results show that CRC-infiltrating DN B cells express lower levels of IgA and higher levels of IgG, compared with healthy tissue B cells (Fig. 1C). Based on data reported in the literature^30^ and our own data, we speculate that a substantial fraction of these cells derive from activated IgM⁺ B cells that have undergone isotype switching toward IgG, potentially contributing to an anti-tumor immune response. The significant reduction of IgA⁺ DN B cells observed in tumor samples further supports the hypothesis of a shift from primary mucosal surface protection mediated by IgA⁺ B cells toward IgG⁺ tumor-reactive B cells. However, we also observe an accumulation of DN B cells in the tumor tissue, characterized by low expression of CD21, a phenotypic marker associated with exhaustion^12^(Fig. 1B), along with a reduced responsiveness of B cells to differentiation stimuli (Fig. 1E). We hypothesize that tumor-infiltrating B cells may be exposed to nutrient competition and/or chronic antigen stimulation, which may drive their dysfunction and eventual exhaustion, despite the initial attempt to respond through IgG class switching. Consistent with this hypothesis, in vitro results demonstrate metabolic reprogramming in B cells towards increased mitochondrial respiration during the early phases of interaction with tumor organoids (Fig. 6A and B), which is likely aimed at providing energy to support the immune response. However, B cells are unable to sustain the increased mitochondrial activity for an extended period (Fig. 6A and B), despite maintaining higher mitochondrial dependence compared with those cultured with healthy organoids (Fig. 5A).

In contrast, B cells co-cultured with tumor and healthy organoids have a similar glucose dependence (Fig. 4A). However, their glucose uptake capacity is significantly reduced in the presence of tumor organoids (Fig. 4C), resulting in a decreased glucose uptake (Fig. 4D). This may explain why B cells maintained with tumor organoids—though equally dependent on glucose—exhibit a lower glycolytic rate compared with those co-cultured with healthy organoids (Fig. 4E and F). It is therefore conceivable that they try to compensate for the deficiency of energy deriving from glucose by increasing their dependence on mitochondria in an attempt to utilize other substrates to restore ATP production by OXPHOS. However, a reduced glycolytic rate leads to a lower production of TCA cycle intermediates, resulting in decreased generation of reducing equivalents (such as NADH and FADH₂) that are essential for OXPHOS. Furthermore, a reduction in uptake capacity of FA—an alternative source of TCA cycle substrates—was observed (Fig. 7C), along with a general downregulation of enzymes involved in FAO (Fig. 7D). As a result, it is plausible that B cells, despite exhibiting increased mitochondrial dependence (Fig. 5A), also display a reduced mitochondrial respiration rate (Fig. 6A, B, C and D), in addition to a decreased glycolytic rate (Fig. 4E and F).

Since the culture method used primarily allows the assessment of interactions between B cells and organoids in terms of soluble factors (Supplementary Fig. S3), we speculate that the observed effects on the metabolism of B cells co-cultured with tumor organoids are independent of direct cell–cell contact. This would also support the hypothesis that nutrient competition may contribute to the metabolic attenuation observed, alongside other tumor-derived factors. Identifying these factors could provide critical insights into the interplay between B cells and the tumor, highlighting opportunities to modulate the TME and strengthen B cell function.

Although these aspects still require in vivo validation, this study provides the first evidence that B cell metabolism is profoundly shaped by tumor cells, acquiring characteristics that differ markedly from those observed in B cells residing in a healthy intestinal environment (Fig. 3D). We are aware, however, that our work has inherent limitations: it offers a predominantly descriptive snapshot of B cell metabolic states within the CRC TME and does not yet elucidate the mechanistic pathways that could enable metabolic restoration and, consequently, impact the functional roles B cells might exert in this context. Nevertheless, by uncovering previously unrecognized metabolic features of B cells in the CRC TME, our study lays an important foundation for future mechanistic investigations and opens valuable avenues toward the development of novel immunometabolic therapies.

## Methods

### CRC biopsies

Biopsies of stage II and stage III tumors and adjacent normal tissues were provided by the Department of General Surgery at Ospedale Universitario “S. Maria della Misericordia” (ASUFC). 2–2.5 g of tumor tissue were reduced into 2 mm pieces. 2–2.5 g of the adjacent normal tissue were cleared of blood vessels and adipose tissue and then reduced into small pieces. Pieces derived from both tissues were then moved in DMEM/F12 medium with 100 U/ml Penicillin, 100 mg/ml Streptomycin, 0.4mM L-Glutamine (EuroClone), 10mM Hepes (EuroClone) and containing 100 mg/ml Primocin™ (InvivoGen), 50 µg/ml Liberase (Sigma-Aldrich) and 10 μm Y-27,632 dihydrochloride (ProdottiGianni) and maintained for 1 h at 37 °C under gentle shaking to allow tissue digestion. The digested solutions were then filtered through a 100 μm cell strainer to obtain a single-cell suspension for analysis.

## Flow cytometry

For flow cytometry analyses, cells were stained for 25 min at 4 °C in the dark with fluorochrome-conjugated monoclonal antibodies or with fluorescent molecules (see Supplementary Tables 2, 3), following manufacturer instructions, according to standardized methods^31^. Thereafter, samples were washed with PBS and acquired with an Attune NxT flow cytometer (ThermoFisher, Eugene, OR). Data were then analyzed by using FlowJo software.

### ASC expansion

B cells sorted from normal and tumor tissues were stimulated with 0.35 μm CpG^16^ and 50ng/ml IL-2^17^ and ASC expansion was assessed after 6 day of stimulation by analyzing the percentage of CD138^+^ cells.

### Mice

Female wild-type C57BL/6 mice (8–12 weeks old, with an average body weight of 19.5 g ± 1.2 g) were purchased from Envigo (Netherlands) and housed in the animal facility of the Dipartimento di Medicina, Università degli Studi di Udine, Udine, Italy.

### AOM/DSS treatment for the induction of colitis-associated colon cancer

Colitis-associated colon cancer was induced in 8-week-old C57BL/6 mice by a single intraperitoneal injection of the mutagen azoxymethane (AOM) (Sigma-Aldrich) (10 mg/kg body weight, diluted in PBS) followed by 3 cycles of dextran sulfate sodium salt (DSS) (MP Biomedicals) (MW 36,000–50,000 at 2.5% in drinking water). Each cycle was followed by 14 days of recovery with normal drinking water. The mice had an average body weight of 19.5 g ± 1.2 g at the start of the treatment. During the treatment, body weight was monitored daily and compared with that of the control group. At the end of the treatment, a significant reduction in body weight of approximately 10% was observed. Mice were then euthanized by cervical dislocation in accordance with institutional and national ethical guidelines and approved protocols.

### Murine organoid generation and culture

Healthy intestinal organoids were generated from isolated crypts of murine healthy small intestines. The intestine was extracted, and the lumen was washed with PBS, 100 U/ml Penicillin, and 100 mg/ml Streptomycin. The intestine was opened longitudinally and the lumen was scraped to eliminate the villi and the excessive mucus. The tissue was sliced into small pieces which were then incubated in PBS, 100 U/ml Penicillin, 100 mg/ml Streptomycin, 0,5 mM DTT (di-thiotreithol), and 5 mM EDTA for 1 h at 4 °C in gentle shaking. The pieces were then resuspended in PBS, 100 U/ml Penicillin, 100 mg/ml Streptomycin and 0,5 mM DTT (di-thiotreithol) to be vigorously pipetted to induce the release of the crypts. Crypts enriched supernatant was collected and the process was repeated four more times. Crypts were centrifuged at 800 rpm for 3 min at 4 °C and resuspended in cold Advanced DMEM/F12 with 100 U/ml Penicillin, 100 mg/ml Streptomycin, 0.4mM L-Glutamine and 10mM Hepes, mixed with Matrigel (Corning) with 1:2 ratios and plated into pre-warmed 24 well Greiner plates (three 10 µl drops/well). Crypts were maintained in WENR medium (Supplementary Table 4). Tumor organoids were generated from colon adenomas induced following the AOM/DSS protocol as described in Sato et al., 2009. Once isolated from the colon, adenomas were processed as described for a healthy intestine, to obtain a suspension of single tumoral cells that were then embedded in Advanced DMEM/F12 with 100 U/ml Penicillin, 100 mg/ml Streptomycin, 0.04mM L-Glutamine, and 10mM Hepes and Matrigel (Corning) and seeded in ENR medium (Supplementary Table 4). Organoids were passed twice a week.

### B cell purification and culture

B cell isolation kit (Miltenyi) was used to purify B cells from the spleens of 8- to 12-week-old mice with an average body weight of 19.5 g ± 1.2 g, euthanized by cervical dislocation in accordance with institutional and national ethical guidelines and approved protocols. Purified B cells were cultured in RPMI at a final concentration of 1 × 10^6^ cells/mL. For B cell stimulation, 10 µg/mL LPS (Sigma-Aldrich) was added to the medium.

### B cell-organoid co-cultures

To set up co-cultures, B cells were resuspended at the final concentration of 1*10^6^/ml in ENR medium (Supplementary Table 4) supplemented with 10 µg/mL LPS (Sigma-Aldrich). 0.5*10^6^ were seeded in each well of organoids, passed 24 h before. Co-cultures were maintained for 24–48 h. Thereafter, B cells were collected and analyzed.

### QSM

For the metabolic analysis, we employed the Quantitative System Metabolism (QSM) pipeline developed by Doppelganger Biosystem GmbH. QSM data analysis uses quantitative information based on the expression levels of metabolic proteins to determine metabolic fluxes and capacities. QSM dataset is reported in Supplementary dataset file.

### Functional Profile of Energy Metabolism with Single-Cell Resolution (SCENITH)

SCENITH assay was performed using the kit kindly provided by the laboratory of professor Andrea Cossarizza (University of Modena and Reggio Emilia, Modena, Italy) and following the protocol described by Arguello et al.^19^, with appropriate changes^32^. Briefly, following co-culture, B cells were divided into four conditions with 6*10^6^ cells each, resuspended in complete RPMI media and incubated with control (DMSO) or metabolic inhibitors (100mM 2-DG; 1mM Oligomycin; the sequential combination of the drugs at the same concentrations). 10 µg/ml Puromycin was immediately added in all conditions and the treatments were maintained for 40 min at 37 °C. Next, cells were washed and stained for surface and viability markers, as described in the “Flow cytometry” section of Materials and Methods. Samples were then fixed and permeabilized with FOXP3 fixation and permeabilization kit (ThermoFisher) following manufacturer instructions and stained for Puromycin with anti-Puromycin Alexa Fluor 647 for 1 h at RT. SCENITH was performed on B cells following 48 h of co-culture with the organoids.

### Mitochondrial bioenergetics and metabolic assays

The bioenergetic profile of B cells was evaluated with the XFe96 Extracellular Flux Analyzer (Seahorse Bioscience), as described in^33^. For the evaluation of glycolytic activity, B cells were harvested after co-culture and washed with Agilent Seahorse XF Base Medium Minimal DMEM supplemented with 1mM L-Glutamine (ECAR -extracellular acidification rate- medium). To assess mitochondrial respiration, B cells were washed with Agilent Seahorse XF Base Medium Minimal DMEM supplemented with 1mM sodium pyruvate, 10mM glucose, and 1mM L-Glutamine (OCR medium). Cells were then resuspended at a concentration of 4*10^5^ cells in 175µL of ECAR or OCR medium and plated into an XF96 Cell Culture Microplate that was centrifuged for 3 min at 250 g to allow cells to settle at the bottom. Then, the plate was incubated for 1 h at 37 °C in an incubator without CO_2_. Real-time measurements of ECAR were made in the XFe96 extracellular flow analyzer following the addition of the interrogating drugs, 80mM glucose, 18µM Oligomycin, and 1 M 2-Deoxy-D-Glucose (2-DG). OCR was measured following the addition of 16µM oligomycin, 13.5µM fluoro-carbonyl cyanide phenyl hydrazone (FCCP), 10µM antimycin A (Ant), 10µM and rotenone (Rot). Wave desktop software was used for the analysis.

### Western blot analysis

0.35*10^6^ B cells per experimental condition were used to prepare the total cell lysate. Cell pellets were lysed in lysis buffer (25 mM Tris-HCl [pH7.4], 150 mM NaCl, 1 mM EDTA, 1% NP-40, 5% glycerol, 1 mM Na3VO4, 50 mM NaF (Sigma Aldrich), and Complete Mini protease inhibitor cocktail (Roche, according to manufacturer instructions)) for 20 min on ice. Lysates were then centrifuged at 12,000 rpm for 20 min at 4 °C. BCA™ Protein Assay Kit (Pierce) was used to determine protein concentration. Lysates were then diluted with 4x Laemmli buffer and denatured at 95 °C for 10 min. For the analysis of ETC complexes subunits, samples were denaturated at 65 °C for 15 min. Samples were separated on SDS 10% polyacrylamide gels and blotted on nitrocellulose membranes (Amersham). Then, membranes were incubated in Tris-buffered saline (TBS) containing Tween 20 (0.05%) and Bovin Serum Albumin (BSA) (5%) at RT for 1 h to block the nonspecific binding sites and then incubated with primary antibodies. Antibodies were then washed three times in PBS. Hexokinase II, PKM, Enolase I and ERK were detected by incubating respective membranes with a peroxidase-conjugated secondary antibody according to manufacturer instructions (Amersham Biosciences). Peroxidase activity was detected with the ECL system (Amersham Biosciences) or Femto (Pierce). The densitometric analysis of the bands was performed with ImageStudio Software. To detect ATP5A, UQCRC2, SDHB, COX II and CS following incubation with respective primary antibodies, membranes were incubated with specific fluorescent-conjugated secondary antibodies (Anti-Mouse IgG (H + L) CF770 -Sigma Aldrich, catalog number SAB4600214- for ATP5A, UQCRC2, SDHB and COX II; Anti-Rabbit IgG (H + L) 680RD -LI-COR, catalog number 926–68071- for CS) and developed with the NIR Fluorescence technology (LI-COR Biosciences). Following acquisition, the densitometric analysis of the bands was performed by Odyssey CLx Infrared Imaging System (LI-COR Biosciences). Antibodies used are listed in Supplementary Table 5.

### Statistics

Statistical analyses were performed using GraphPad Prism 8. Two-sided non-parametric tests were applied, assuming a non-normal distribution of the data –except for Seahorse data–: the Mann–Whitney test was used for comparisons between two groups, and the Kruskal–Wallis test for comparisons among three or more groups. Seahorse data were analyzed using a two-sided two-way ANOVA. Results are presented as mean ± SD, and P values < 0.05 were considered statistically significant.

### Study approval

The studies involving humans were approved by Comitato Etico Unico Regionale per il Friuli Venezia Giulia (CEUR FVG), Prot n. 4365 dd 01/02/2024. The studies were conducted in accordance with the local legislation and institutional requirements. The participants provided their written informed consent to participate in this study. Animal housing and experimentation were performed in adherence to ARRIVE Guidelines and conducted in compliance with Italian legislation (D.lgs 26/2014). In vivo experiments were approved by the Italian Ministry of Health (authorization numbers 634/2015-PR).

## Supplementary Information

Below is the link to the electronic supplementary material.

Supplementary Material 1

Supplementary Material 2

Supplementary Material 3

Supplementary Material 4

## Data Availability

All data generated or analysed during this study are included in this published article (and its Supplementary Information files).
