# Supplementary material for: Immunophenotypic skewing of B cells toward IgD⁻CD27⁻IgG⁺ subtype and metabolic attenuation in colorectal cancer
Source: Sci Rep. 2026 Feb 28;16:11403. doi: 10.1038/s41598-026-41446-x (PMC13056914; doi:10.1038/s41598-026-41446-x)
Supplement: Supplementary file 1 — Supplementary Material 1 [file 41598_2026_41446_MOESM1_ESM.pdf]

Supplementary figures

S1

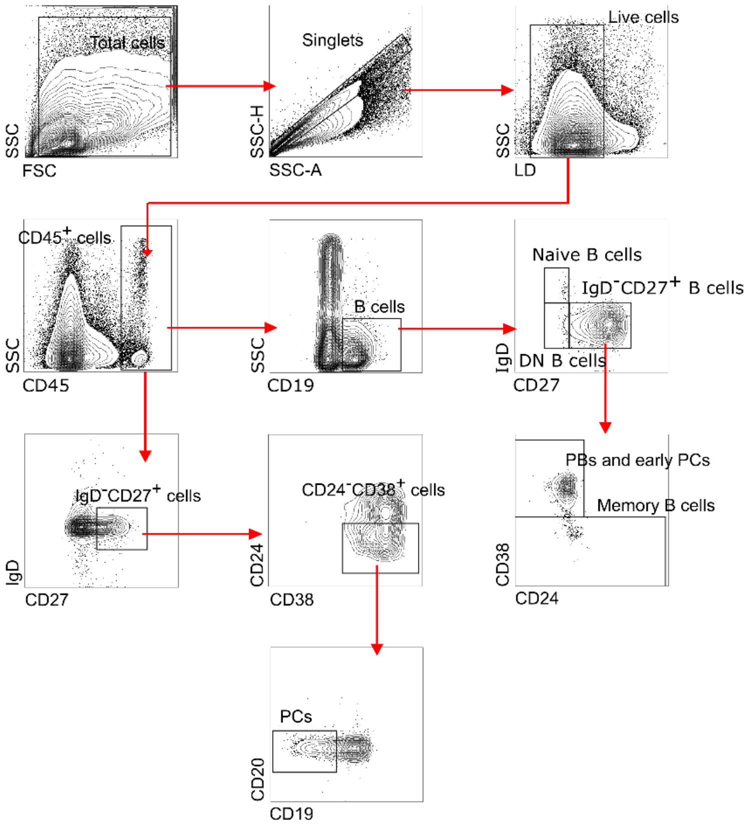

S1. Flow cytometry gating strategy for B cell immunophenotypic profile. Representative flow cytometry gating strategy used to analyze B cell immunophenotypes in healthy and tumor tissues is illustrated.

S2

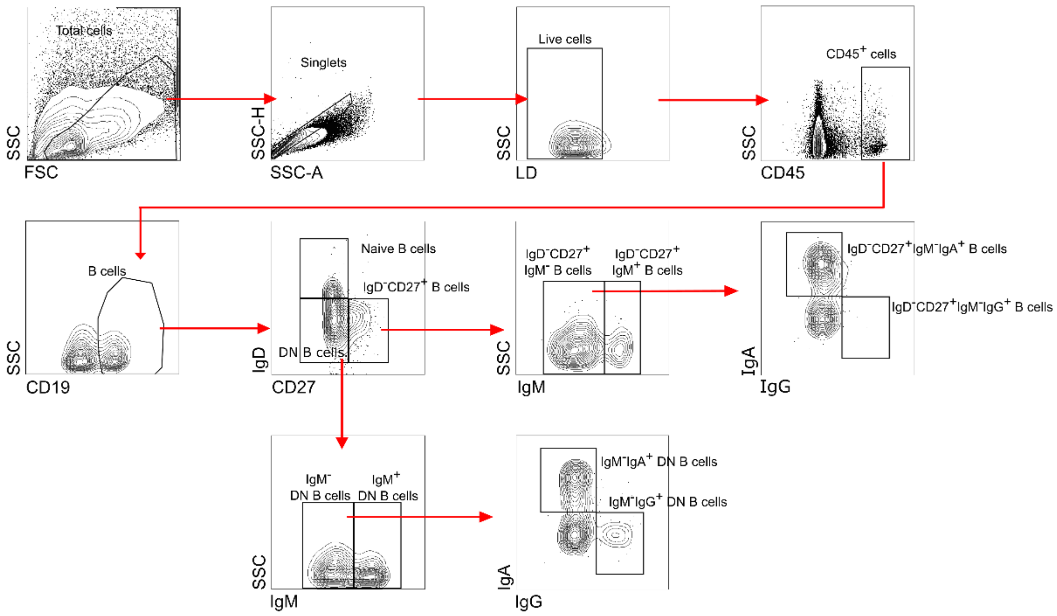

S2. Flow cytometry gating strategy for B cell immunoglobulin profile. Representative flow cytometry gating strategy used to analyze B cell immunoglobulin profile in healthy and tumor tissues is illustrated.

S3

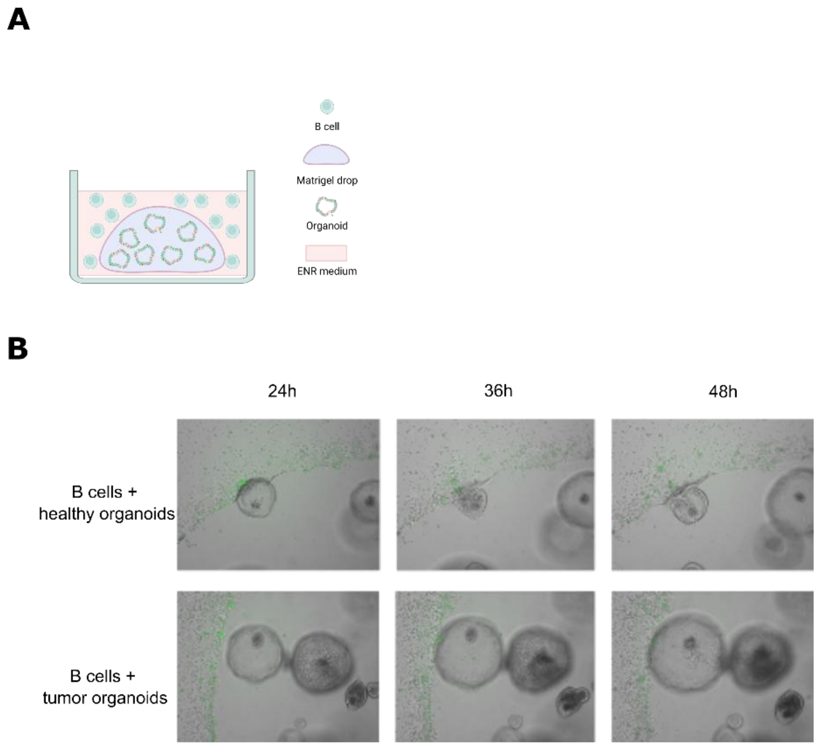

S3. Co-culture system between murine B cells and organoids. A. Graphical representation of organoids embedded in Matrigel drops, co-cultured with B cells in suspension in ENR culture medium (see Supplemental Table 3 for ENR composition). **B.** Time frames of the time-lapse experiment by fluorescence microscopy of B cells + healthy organoids and B cells + tumor organoids, labeled with CellTrace CFSE (Thermo Fisher), at 24-, 36- and 48-hour time-points.

S4

A

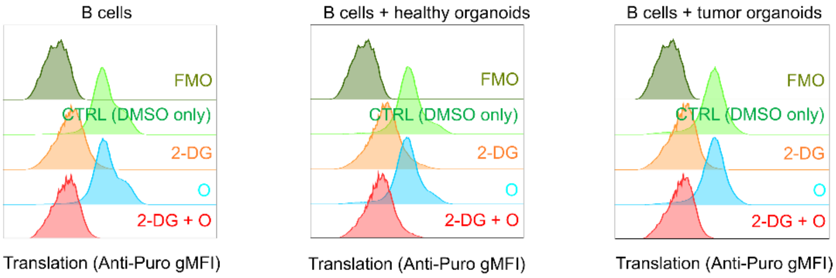

B

Glucose dependence (%): 100*((CTRL - 2-DG)/(CTRL - 2-DG+O))

Mitochondrial dependence (%): 100*((CTRL - O)/(CTRL - 2-DG+O))

FAO AAO dependence (%): (100-(100*((CTRL - 2-DG)/(CTRL - 2-DG+O))))

S4. Metabolic monitoring using SCENITH. A. Representative histograms showing the gMFI of Anti-Puromycin antibody in B cells alone or co-cultured with healthy and tumor organoids, following 2-deoxyglucose (2-DG), Oligomycin (O) and 2-DG + O treatments. **B.** Glucose, mitochondrial and AAO and FAO dependences were calculated from the gMFI of Anti-Puromycin antibody upon the treatments following the formulas.

S5

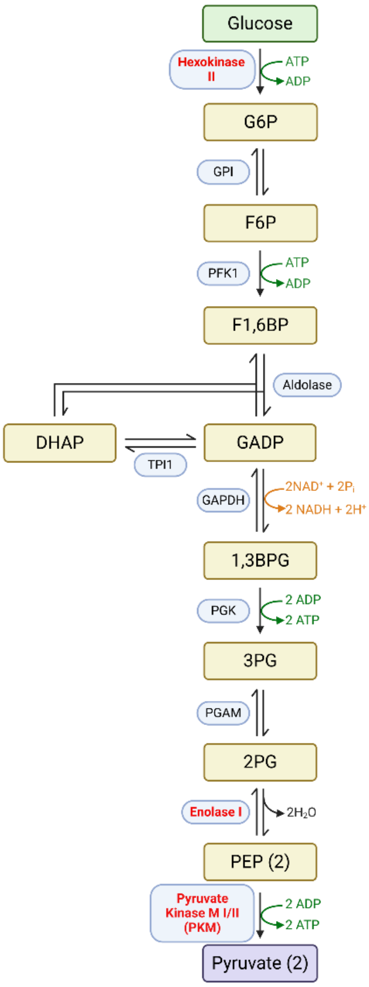

S5. Schematic representation of the glycolytic pathway. Key glycolytic enzymes tested in this study are highlighted in red. Created in https://BioRender.com.

S6

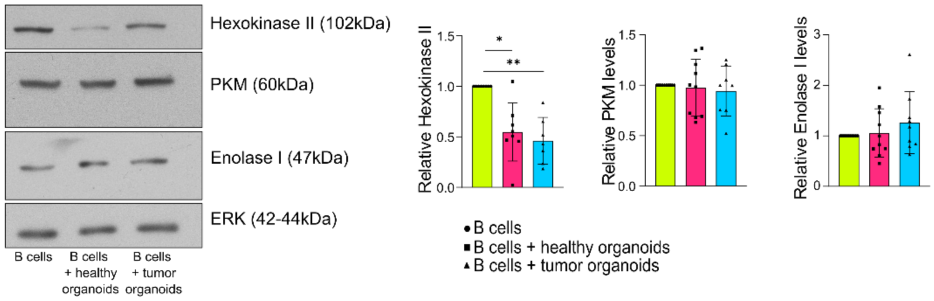

S6. Protein expression levels of glycolytic enzymes. Western blot analysis of Hexokinase II, PKM I/II and Enolase I in B cells alone and after co-culture with healthy and tumor organoids (cropped blots are shown; uncropped images are available in the Supplementary Fig. S7-S10); the densitometry analysis was calculated over ERK signal and expressed in fold induction relative to B cells alone. Statistical analysis was performed with Kruskal-Wallis test. *=$p<0.05$ **=$p<0.01$, ***=$p<0.001$ ($n\geq8$).

S7

Hexokinase I (102kDa)

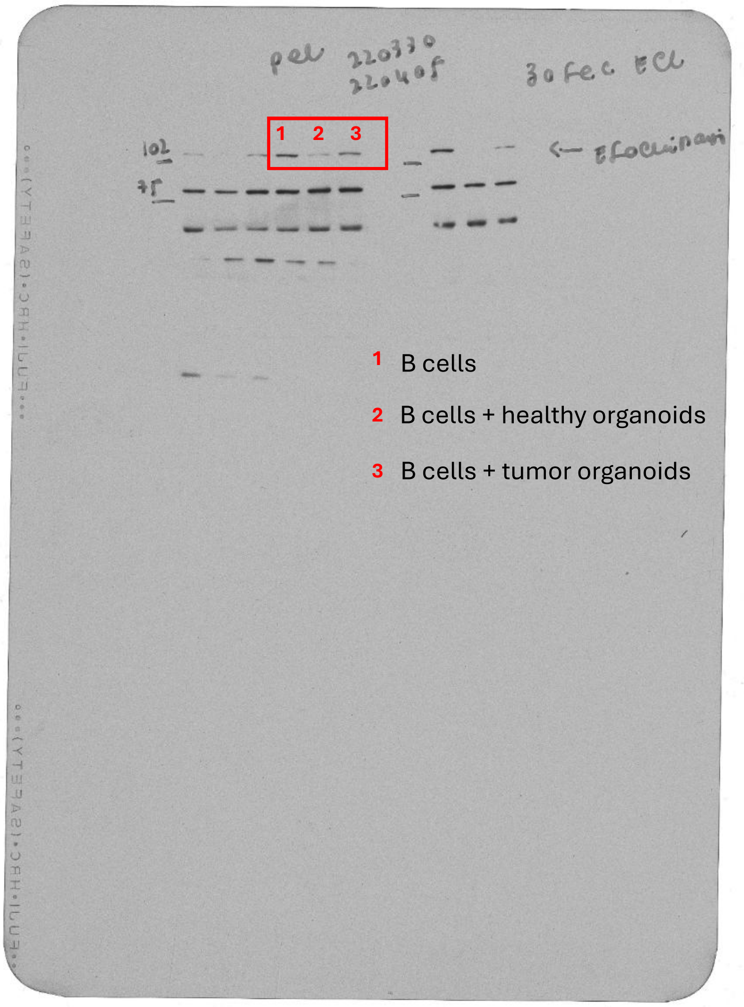

S7. Uncropped Western blot analysis of Hexokinase II (102kDa).

S8

PKM (60kDa)

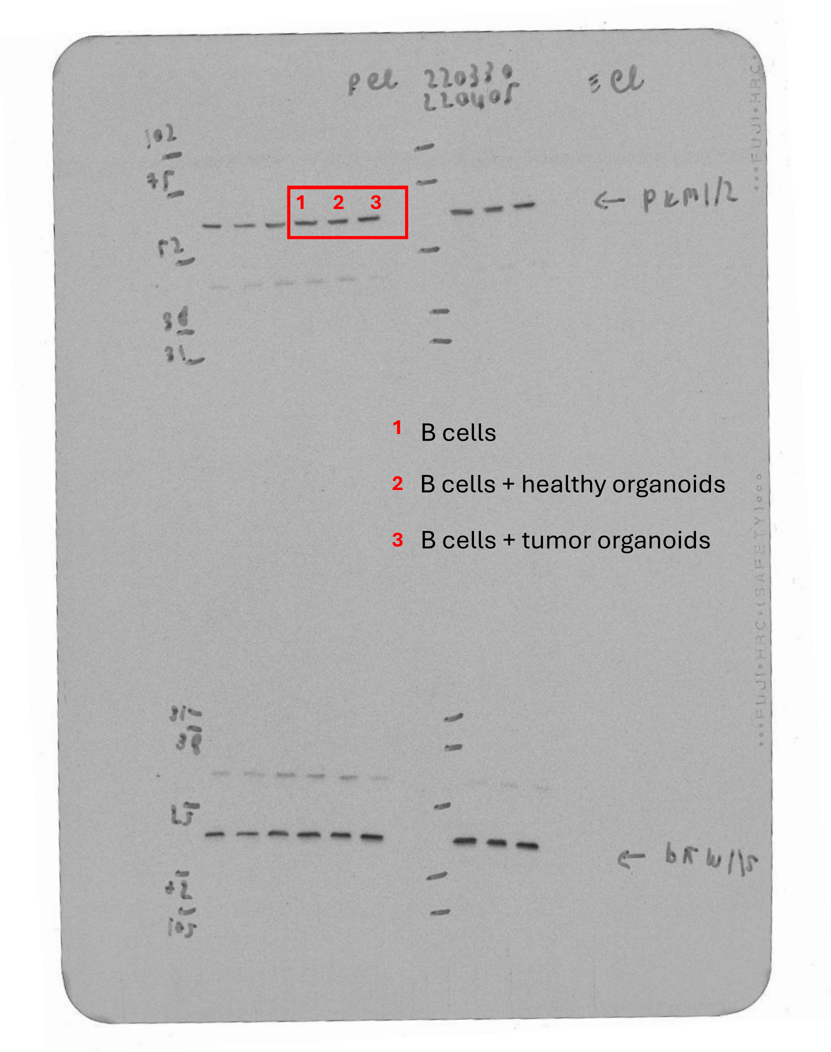

S8. Uncropped Western blot analysis of PKM I/II (60kDa).

S9.

Enolase I (47kDa)

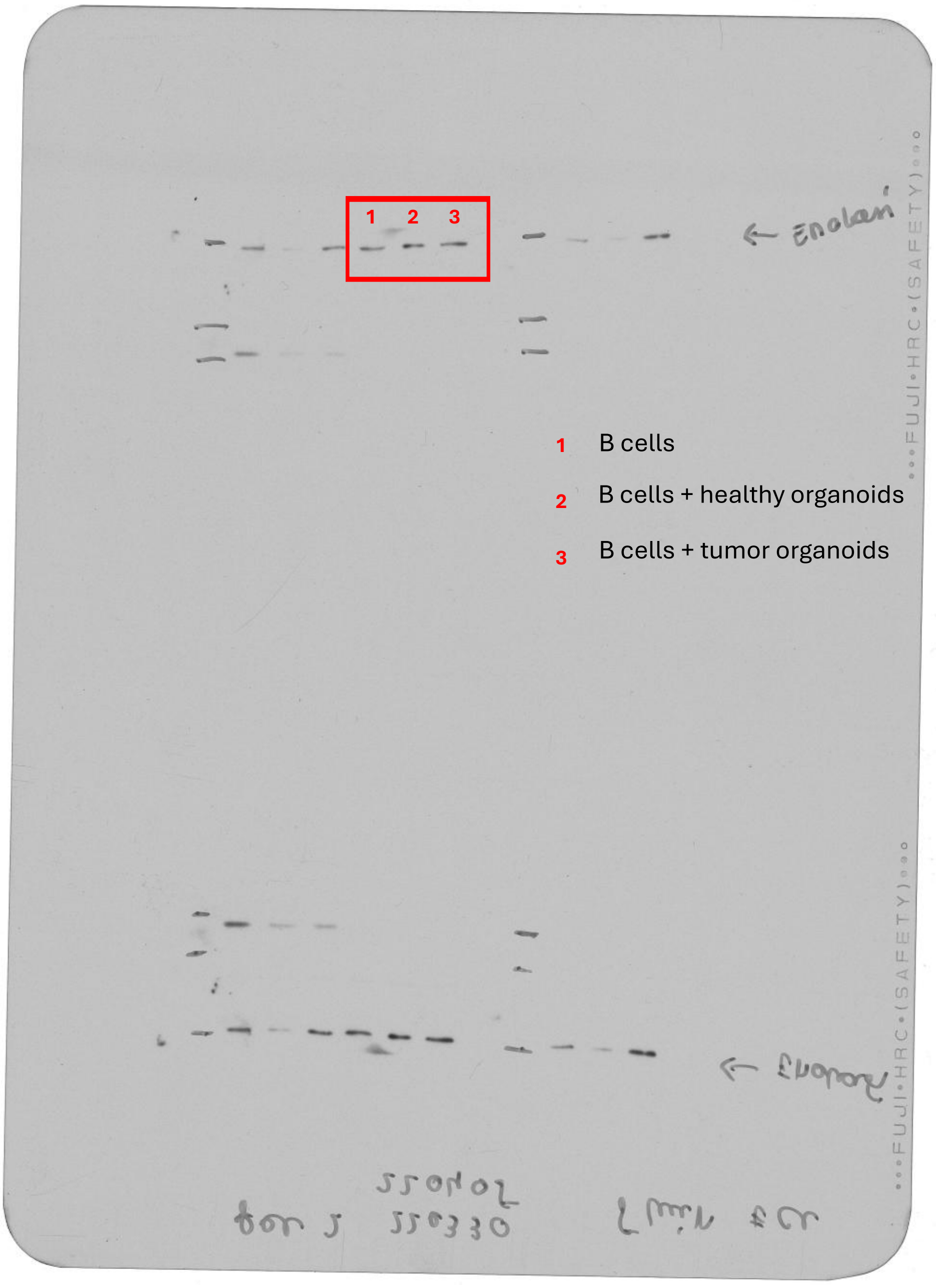

S9. Uncropped Western blot analysis of Enolase I (47kDa).

S10

ERK (42-44kDa)

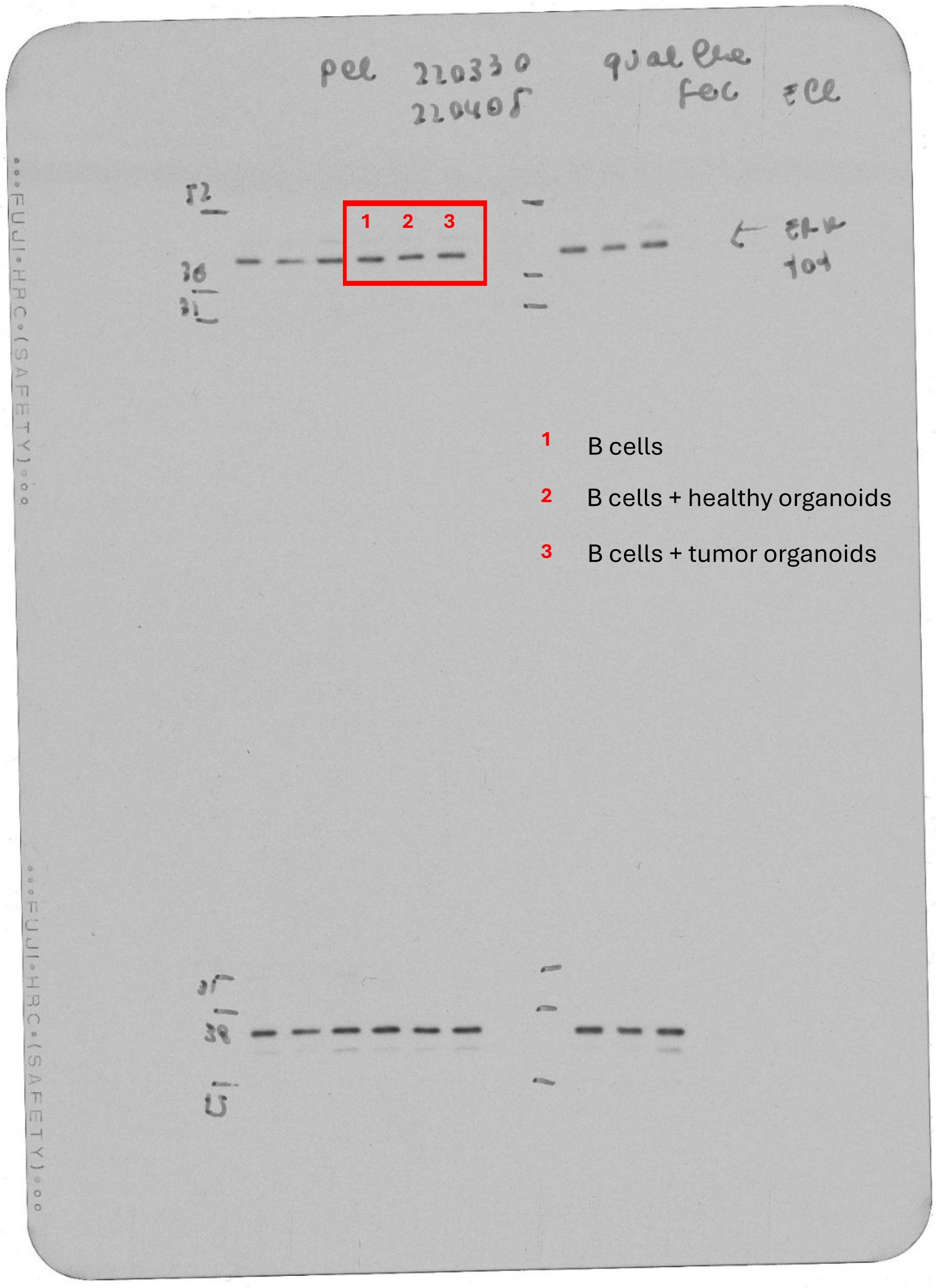

S10. Uncropped Western blot analysis of ERK (42-44kDa)

S11

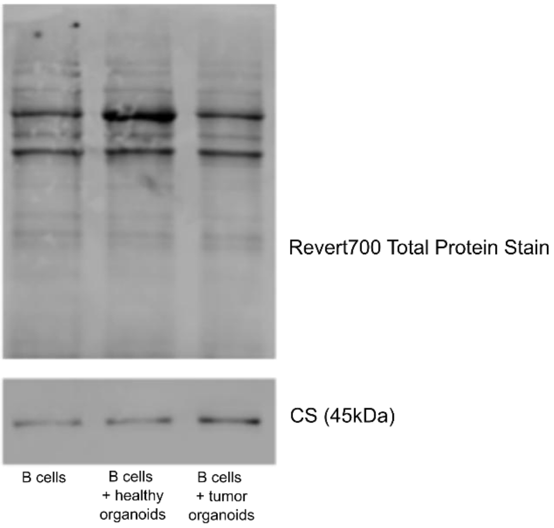

S11. Revert700 Total Protein Stain signal. The Revert700 Total Protein Stain signal was used to normalize the protein expression levels of CS in B cells alone and after co-culture with healthy and tumor organoids (cropped blots are shown; uncropped images are available in the Supplementary Fig. S13, S14).

S12

OXPHOS subunits

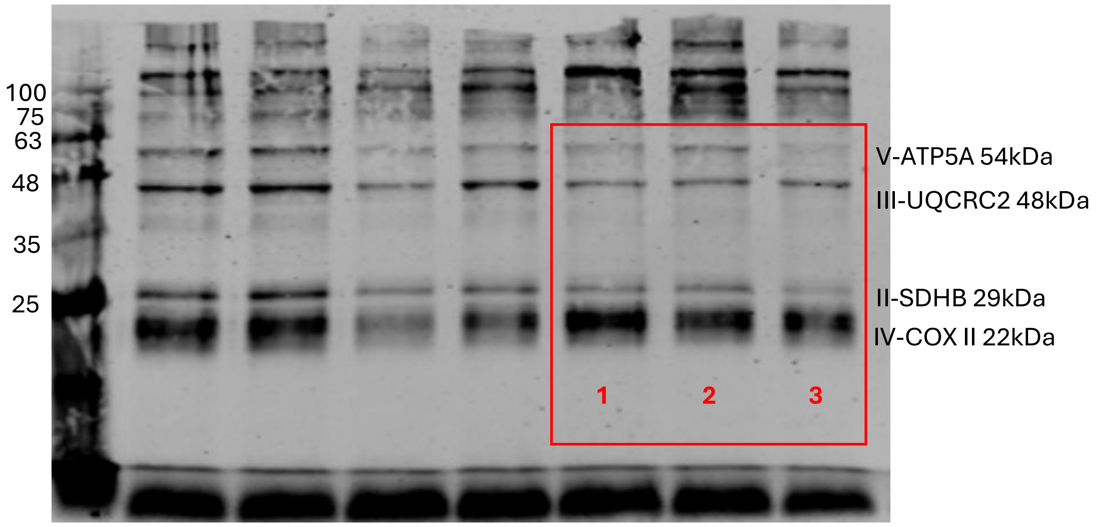

1 B cells

2 B cells + healthy organoids

3 B cells + tumor organoids

S12. Uncropped Western blot analysis of OXPHOS subunits.

S13

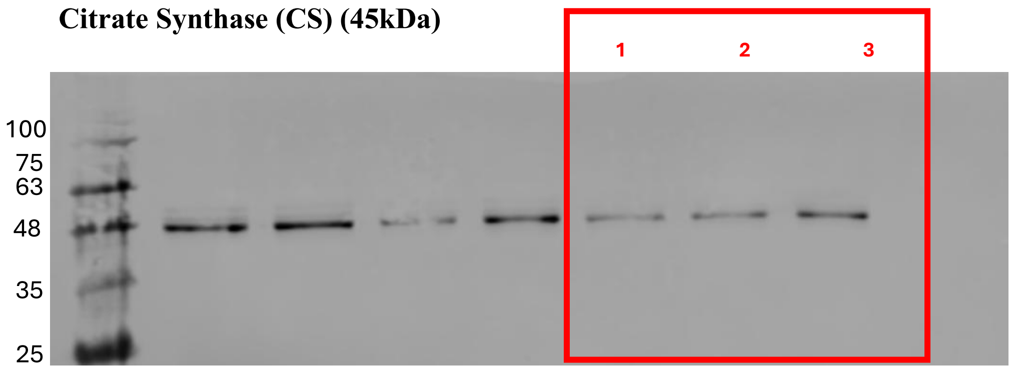

1 B cells

2 B cells + healthy organoids

3 B cells + tumor organoids

S13. Uncropped Western blot analysis of CS (45 kDa).

S14

Revert700 Total Protein Stain

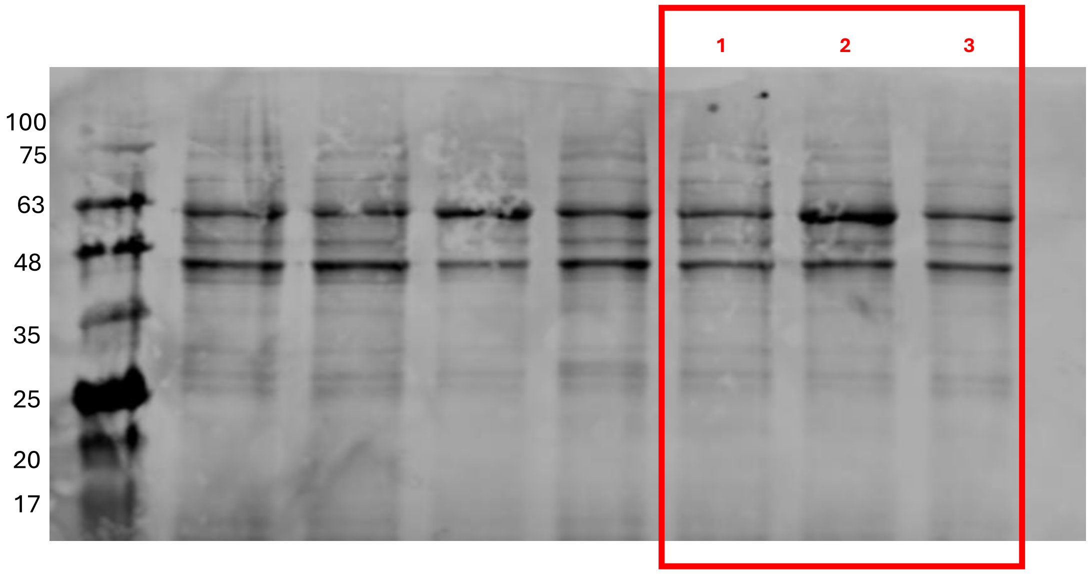

1 B cells

2 B cells + healthy organoids

3 B cells + tumor organoids

S14. Uncropped Western blot analysis of Revert700 Total Protein Stain.

S15

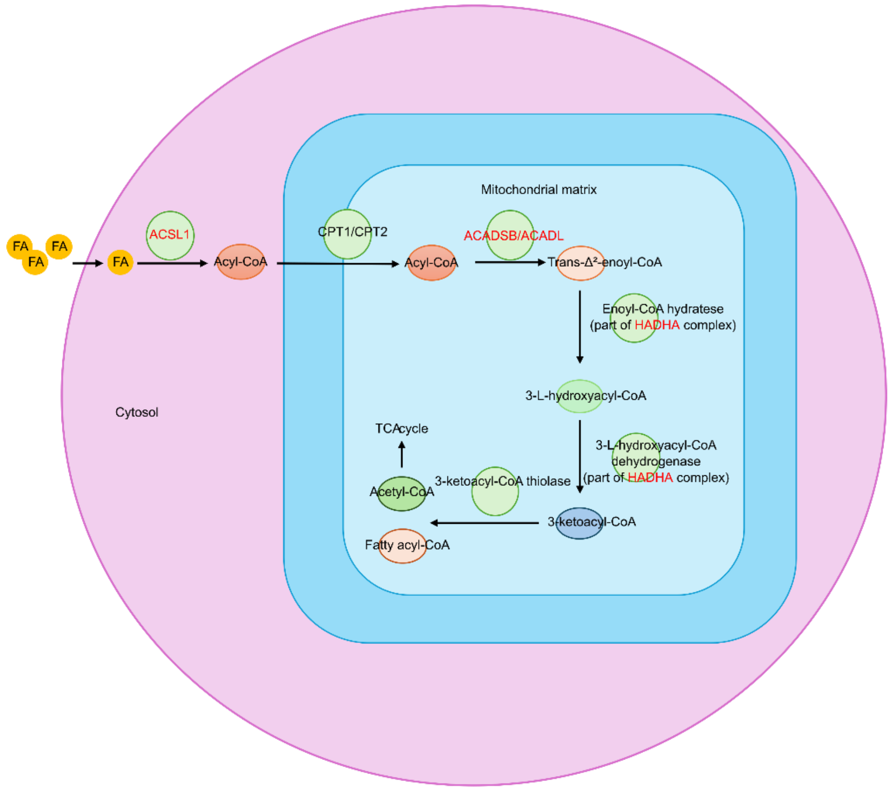

S15. Schematic representation of the FAO pathway. Selected FAO enzymes tested in this study are highlighted in red.

Supplementary Tables

Table 1. Fluorescent conjugated antibodies

Description	Company	Catalog number
Anti-hCD45 FITC	BioLegend	304006
Anti-hCD19 APC	BioLegend	302212
Anti-hCD20 BV510	BioLegend	302339
Anti-human CD24 Pe-Dazzle594	BioLegend	311133
Anti-hCD27 PE-Cy7	Invitrogen	25-0279-42
Anti-hCD38 BV421	BioLegend	303525
Anti-hIgM PerCP-Cy5.5	BioLegend	314511/2
Anti-hIgD SB702	Invitrogen	67-9868-42
Anti-hCD138 Pe-Dazzle594	Sony	2382650
Anti-hCD21 SB600	Invitrogen	63-0219-42
Anti-hIgA PE	Miltenyi	130-113-476
Anti-hIgG BV421	Sony	2653515
Anti-Puromycin	Provided by the laboratory of Professor Andrea Cossarizza	
Anti-mCD19 APC Fire 750	BioLegend	115557
Anti-mCD138 PE	BD Pharmingen	553714

Table 2. Fluorescent molecules

Live/Dead (LD) eFluor780	Invitrogen	65-0865-14
Live/Dead (LD) Fixable Lime 506	Invitrogen	L34990 A
Cell Trace CFSE	Invitrogen	C34554
2-NBDG	Invitrogen	N13195
TMRM	Invitrogen	T668

Table 3. Composition of organoid culture media

Reagent	Company	Catalog number	WENR	ENR
Advanced Dulbecco's Modified Eagle Medium/F12	Life Technologies	12634028		
B-27® Supplement (50X), serum free	Life Technologies	17504044	1X	1X
N-acetylcysteine	Sigma Aldrich	A9165-25G	1.25mM	1.25mM
Y-27632 dihydrochloride	Prodotti Gianni (Abcam)	120129	10µM	10µM
Primocin™	Aurogene (InvivoGen)	ant-pm-1	100µg/ml	100µg/ml

m-EGF	DBA (PEPROTECH)	315-09	50ng/ml	50ng/ml
Noggin conditioned medium*			10%	10%
R-spondin conditioned medium*			20%	20%
Wnt conditioned medium*			50%	

*Recombinant HEK293T were used for Noggin and R-Spondin conditioned media preparation, recombinant L-cells were used for Wnt conditioned medium preparation.

Table 4. Antibodies and reagents for Western blot analyses of mouse proteins

Description	Company	Catalog number
Anti-Hexokinase II	Cell Signaling	2867S
Anti-PKM I/II	Cell Signaling	3190S
Anti-Enolase I	Cell Signaling	3810S
Anti-Citrate Synthase	Invitrogen	3H8L26
Anti-OxPhos Antibody cocktail	Invitrogen	458199
Anti-p44/42 ERK1/2	Cell Signalling	4696
Anti-Mouse IgG (H+L) CF770	Sigma Aldrich	SAB4600214
Anti-Rabbit IgG (H+L) 680RD	LI-COR	926-68071
Revert700 Total Protein Stain	LI-COR	926-11010
